# Are there any new reliable markers to detect renal injury in obese children?

**DOI:** 10.1080/0886022X.2018.1489284

**Published:** 2018-07-23

**Authors:** Özlem Bostan Gayret, Mehmet Taşdemir, Meltem Erol, Hikmet Tekin Nacaroğlu, Oğuzhan Zengi, Özgül Yiğit

**Affiliations:** a Department of Pediatrics, Ministry of Health, Bağcılar Training and Research Hospital, Istanbul, Turkey;; b Department of Pediatrics, Division of Pediatric Nephrology, Koc University Hospital, Istanbul, Turkey;; c Department of Pediatric Allergy, Medipol University Hospital, Istanbul, Turkey;; d Department of Biochemistry, Ministry of Health, Bağcılar Training and Research Hospital, Istanbul, Turkey

**Keywords:** Cystatin C, KIM-1, NGAL, OPN, MMP-9, obese, children

## Abstract

**Aim:** The aim of this study was to examine the serum and urine levels of kidney injury molecule-1 (KIM-1), neutrophil gelatinase-associated lipocalin (NGAL), osteopontin (OPN), matrix metalloproteinase-9 (MMP-9), and serum Cystatin-C to determine the renal effect of obesity in obese children.

**Methods:** Seventy-two obese and 35 non-obese healthy children were included in this study. Blood pressure (BP) was evaluated with office measurement. Creatinine, cystatin C, lipids, fasting glucose, and insulin levels were measured, and homeostasis model assessment -insulin resistance (HOMA-IR) was calculated. The urine albumin/creatinine ratio was calculated. The serum and urine KIM-1, NGAL, OPN, and MMP-9 levels were measured.

**Results:** Serum cystatin-C, triglyceride, and homeostasis model assessment-insulin resistance (HOMA-IR) index were found to be significantly higher in the obese group (*p* = .0001), and high-density lipoprotein (HDL) cholesterol was found to be significantly lower (*p* = .019) in the obese group. No significant differences were found in serum KIM-1, NGAL, OPN or MMP-9 levels between groups (*p* > .05). No significant differences were found in urine KIM-1 and MMP-9 levels (*p* > .05), Urine NGAL, and OPN levels were found significantly higher in obese groups (*p* < .05).

**Conclusions:** According to our results, although serum KIM-1, NGAL, OPN, MMP-9, and urine MMP-9, urine KIM-1 do not appear to be ideal markers to evaluate renal injury in the early period of obesity, the serum levels of cystatin C and urine NGAL, urine OPN can be used as a good marker for assessing the renal effect of obesity which can lead end stage renal disease in pediatric population.

## Introduction

Childhood obesity is quickly becoming an important health problem worldwide. New epidemiological data show that obesity is related to increased risk of renal injury in children [1]. Recent trials showed that pediatric renal patients have significantly higher BMI z-scores than the normal population [2]. These results indicate that obesity is an independent risk factor for CKD in children.

Obesity-related renal disease is asymptomatic and difficult to diagnose, so useful biomarkers are necessary to prevent serious renal conditions in obese children. Known kidney biomarkers, including serum creatinine (sCr) level, blood urea nitrogen (BUN) level, urine albumin/protein ratio, and volume excretion, do not change quickly during the presentation of acute conditions. Candidate biomarkers for kidney injury have been published previously and include cystatin C, kidney injury molecule-1 (KIM-1), neutrophil gelatinase-associated lipocalin (NGAL), and osteopontin (OPN) [3]. Cystatin C is a low-molecular-weight protein which is a member of the cystatin superfamily of cysteine protease inhibitors. Cystatin C is filtered at the glomerulus and not reabsorbed, but metabolized in the tubules. All nucleated cells produce cystatin C with relatively constant amount, and it is suggested that not affected by changes diet [4,5]. The serum cystatin C level may correlate more closely with the GFR than the serum creatinine concentration [6].

The soluble form of KIM-1 is easily detectable in the urine of patients with acute kidney injury (AKI) or cystic kidney disease [7,8]. Neutrophil gelatinase-associated lipocalin is released from injured renal tubular cells in the early stage of acute kidney injury (AKI). Serum and urine NGAL levels are independent predictors of chronic kidney disease (CKD) progression in patients with moderate renal disease [9].

Osteopontin (OPN), also known as secreted phosphoprotein 1 (SPP 1), bone phosphoprotein, sialoprotein 1, uropontin, and early T-lymphocyte activation-1 (Eta-1), is a secreted matricellular protein [10]. In addition to regulating inflammation in adipose tissue, OPN has an important role in the early cellular immune response, bone structure, and soft tissue remodeling. Furthermore, OPN is highly expressed in the tubular epithelium of the renal cortex and the glomeruli of patients with diabetic nephropathy [11].

MMP-9, a member of the MMP family, is the most important degradation enzyme for collagen IV and plays a fundamental role in damaging the integrity of the basement membrane. Presently, researchers have an interest in MMP-9 and kidney diseases, including glomerulonephritis and diabetic nephropathy [12].

In this study, we first aimed to assess the blood and urine levels of cystatin C, KIM-1, NGAL, OPN, and MMP-9 in obese children and healthy controls. Finally, we focused on the reliability of these biomarkers for detecting early renal injury in obese children.

## Methods

### Study design and patients

This single center, observational, cross-sectional, controlled study included 107 children, 72 of these suffered from obesity. between 3 and 16 years of age who were admitted to our pediatric outpatient clinic between April and August 2015. Obese children with infection, metabolic or endocrine disease and children receiving dietary supplementation were excluded from the study. None of the children were using antihypertensive and antilipidemic drugs. The other 35 children were children with normal nutrition applied in routine health checks and who agreed to participate in the study. Medical records were evaluated for age, gender and physical examination findings. Weight and height were measured, and body mass index was calculated. Patients with a BMI greater than the 95th percentile for their age and gender were considered obese.

### Blood pressure measurements

Casual blood pressure (BP) was measured three times by an oscillometric device (Nihon Kohden, Vismo, Germany) and averaged; indexed systolic (S) and diastolic (D) BP were calculated by dividing the mean casual S and D BP values by their corresponding 95th percentile values based on the patient’s height and gender [13].

### Laboratory measurements

Blood samples were collected in the morning after an overnight fast (at least 8 h) for measurements of complete blood count and biochemical parameters, including creatinine (Cr), cystatin C, total cholesterol (TC), low-density lipoprotein cholesterol (LDL-C), high-density lipoprotein cholesterol (HDL-C), triglyceride, glucose, and insulin levels. Homeostasis model assessment-insulin resistance (HOMA-IR) was calculated using the following formula: [Fasting insulin (µU/mL) × Fasting glucose (mg/dL)/405] [14]. Estimated glomerular filtration rate (eGFR) was calculated using the Schwartz formula [15]. Measurement of creatinine was made standardized by SRM 914 and SRM 967 (ID/MS). Cystatin C level was analyzed with Human Cystatin C kit and measured in the reconstituted material by particle enhanced immune-nephelometry, particle enhanced immuno-turbidimetry and single radial immuno-diffusion. For urine sediment examination, at least 10 mL of urine was centrifuged at 3000 rpm for five minutes. Pyuria and hematuria were defined as the presence of 10 or more white cells and 10 or more red blood cells per high-power field, respectively. Microalbuminuria was defined as a urinary albumin/creatinine ratio of 30–300 mg/g. Routine biochemical tests were performed using an automated clinical chemistry analyzer. To assess serum and urinary KIM-1, NGAL, OPN, and MMP-9 levels, blood and urine samples were collected and centrifuged, and the supernatants were frozen at −80 °C until analysis. Enzyme-linked immunosorbent assay (ELISA) was used to measure KIM-1 (Shanghai Sunred Biological Technology Co Shanghai, China, Human KIM-1 kit, Cat No: 201–12-1100), NGAL (Shanghai Sunred Biological Technology Co Shanghai, China, Human NGAL kit, Cat No: 201–12-1720), OPN (Affymetrix eBioscience, San Diego, CA, Human OPN kit, Cat no: BMS2066) and MMP-9 (Shanghai Sunred Biological Technology Co Shanghai, China, Human MMP-9 kit, Cat No: 201–12-0937) levels. The serum and urine KIM-1, NGAL, OPN, and MMP-9 levels were measured.

The present study was approved by the Ethics Committee of Bağcılar Training and Research Hospital (approval number 2015/366) and was conducted in accordance with the Declaration of Helsinki. Study participants and/or their parents gave consent prior to participation.

### Statistical analysis

Statistical analyses in this study were performed using NCSS (Number Cruncher Statistical System) 2007 Statistical Software (UT) package. In the data evaluation, in addition to descriptive statistical methods (mean, standard deviation), the independent *t*-test was used in the comparison of the paired groups of variables exhibiting a normal distribution, the Mann–Whitney U test was used in the comparison of the paired groups of variables that did not exhibit a normal distribution, and the chi-square test was used in the comparison of qualitative data. The results were evaluated at a significance level of *p* < .05.

## Results

As shown in Table 1, age and gender were not significantly different between the obese and non-obese children. The mean ages of the obese and non-obese groups were 9.87 ± 2.85 years and 8.68 ± 3.19 years, respectively. Weight, height, and body-mass index (BMI) were significantly higher in the obese group (*p* = .0001).

**Table 1.** Demographic features of the obese and non-obese groups.

		Non-obese		Obese		*p* value
Age (years)		8.68 ± 3.19		9.87 ± 2.85		.055
Gender	Female	21	60.00%	41	56.94%	.764
	Male	14	40.00%	31	43.06%	
Weight (kg)		28.25 ± 9.37		60.71 ± 22.46		.0001*
Height (m)		1.27 ± 0.18		1.43 ± 0.17		.0001*
BMI (kg/m²)		17.19 ± 2.61		28.56 ± 4.95		.0001*
cSKB (mmHg)		108.2 ± 7.9		120.47 ± 13.79		.0001*
cDKB (mmHg)		62.6 ± 8.83		67.74 ± 9.31		.008*

cSBP: casual systolic blood pressure; cDBP: casual diastolic blood pressure.

Casual SBP and DBP were significantly higher in the obese group (*p* = .0001 and .008, respectively) (Table 1). All children in the non-obese group were normotensive. As shown in Table 2, the obese group showed significantly increased serum cystatin C, triglyceride and HOMA-IR index (*p* = .0001) and significantly decreased HDL-C (*p* = .019). Urinalyses revealed microalbuminuria in 8 (11.11%) obese children.

**Table 2.** Basic biochemical laboratory assessments.

	Non-obese *n* = 35	Obese *n* = 72	*p* value
Serum creatinine (mg/dL)	0.46 ± 0.1	0.49 ± 0.1	.142
eGFR (creatinine based)	118.14 ± 23.33	123.85 ± 18.45	.172
Cystatin C (mg/L)	0.85 ± 0.12	1.07 ± 0.17	.0001*
HOMA-IR	1.99 ± 1.08	4.91 ± 4.21	.0001*
LDL-C (mg/dL)	85.11 ± 26.89	88.67 ± 25.44	.507
HDL-C (mg/dL)	60.49 ± 13.27	52.85 ± 16.55	.019*
Triglyceride (mg/dL)	79.14 ± 29.13	115.48 ± 55.67	.0001*
Total cholesterol (mg/dL)	158.66 ± 22.34	162.03 ± 26.43	.517

eGFR: Estimated glomerular filtration rate; HOMA-IR: Homeostasis model assessment-insulin resistance; LDL-C: Low-density lipoprotein cholesterol; HDL-C: High-density lipoprotein cholesterol.

No significant differences in serum osteopontin, MMP-9, NGAL, KIM-1 and urine MMP-9 and urine KIM-1 were observed between the obese and non-obese groups (*p* > .05). Urine NGAL (*p* = .002), and urine OPN (*p* = .02) were significantly higher in the obese group (Table 3).

**Table 3.** Comparing the levels of serum and urine KIM1, NGAL, MMP9 and OPN between the obese and non-obese groups.

	Non-obese *n* = 35	Obese *n* = 72	*p* value
sOPN (pg/mL)	49.44 ± 27.04	50.46 ± 22.73	.853
	46.21 (25.55–73.3)	48.29 (33.11–60.93)	
sKIM-1 (ng/mL)	1.98 ± 1.56	1.93 ± 2.11	.054
	1.73 (1.15–2.31)	1.24 (0.79–1.91)	
sNGAL (ng/mL)	520.26 ± 511.97	523.48 ± 496.48	.626
	339.81 (196.94–506.37)	339.68 (264.17–501.42)	
sMMP-9 (ng/L)	998.14 ± 434.06	1489.05 ± 1621.51	.780
	961.17 (644.59–1358)	846.1 (658.36–1454.59)	
uKIM-1 (ng/mL)	0.88 ± 0.13	0.93 ± 0.21	.198
	0.88 (0.77–0.94)	0.92 (0.8–1.04)	
uNGAL (ng/mL)	279.28 ± 55.79	312.74 ± 50.87	.002*
	303.77 (285.35–331.47)	269.46 (234.18–329.61)	
uMMP-9 (ng/L)	894.92 ± 128.56	91.53 ± 143.38	.775
	881.36 (821.69–999.19)	891.33 (804.53–1002.26)	
uOPN (pg/mL)	210.75 ± 119.81	299.89 ± 202.73	.02*
	215.24 (139.82–230.54)	271.71 (82.47–507.88)	

s: serum; u: urine; OPN: osteopontin; KIM-1: kidney injury molecule-1; NGAL: neutrophil gelatinase-associated lipocalin; MMP-9: matrix metalloproteinase-9.

## Discussion

In the present study, hypertension and insulin resistance (low insulin sensitivity) were frequently observed in the obese group. While the levels of serum KIM-1, NGAL, OPN MMP-9 and urine KIM-1, urine MMP-9 were not significantly different between the groups, the level of serum cystatin C, urine NGAL, and urine OPN were significantly higher in obese participants.

The prevalence of hypertension in childhood is increasing, especially among young children, and this increase is a result of weight gain [16]. Hypertension and diabetes are important risk factors for renal injury in obese children and can lead to end-stage renal failure [17,18].

The association between obesity and hypertension is well-known [19]. Freedman et al. stated that systolic and diastolic BP are 4.5 and 2.4 times higher, respectively, in overweight children compared to normal weight children [20,21]. Hyperinsulinemia, which occurs due to peripheral insulin resistance and hyperleptinemia (due to the presence of increased adipose tissue), has been proposed to be responsible for the over activation of these systems [22,23]. Low insulin sensitivity is a well-known independent contributor to high blood pressure in children [21,24]. Insulin-mediated effects on renal sodium reabsorption and the sympathetic nervous system have been suggested as the main mechanisms underlying the association between reduced insulin sensitivity and increased blood pressure [25]. In the present study, IR group had higher causal SBS levels.

New biomarkers are necessary for early diagnosis of obesity-related renal damage and determining necessary interventions. For this purpose, tubular injury biomarkers, such as kidney injury molecule-1 (KIM-1), N-acetyl-β-D-glucosaminidase (NAG), neutrophil gelatinase-associated lipocalin (NGAL), and netrin-1, were recently investigated in patients with obesity [26,27].

Serum and urinary NGAL levels have been shown to be independent predictors of chronic kidney disease progression in patients with moderate kidney disease [9]. Furthermore, urine NGAL level serves as an early biomarker of diabetic nephropathy [28]. Wang et al. showed that NGAL is an inflammatory marker that is closely related to obesity and the metabolic complications of obesity [29]. In their studies conducted on mice and adult humans, Wang et al. found that serum NGAL levels were significantly higher in those with obesity-related metabolic and cardiovascular complications [29]. NGAL is released from injured renal tubular cells in the early stage of AKI [9]. In another study, NGAL level has also been reported to be significantly higher in obese mice [30]. Göknar et al. demonstrated the level of urinary NGAL was not significantly different between obese and non-obese group [26]. In the present study, urinary NGAL levels significantly increased in the obese participants. Our study results are supported that increased urinary NGAL level is compatible with AKI in early period of pediatric obesity.

Under normal conditions, KIM-1 is absent in the urine; however, it is increased after the development of the following conditions: acute renal failure (caused by ischemia or nephrotoxicity), urolithiasis (particularly in patients who underwent surgical treatment), and cystic kidney disease [8,31,32]. Kidney injury molecule-1 expression was found to be significantly associated with glomerular disease and interstitial damage [33]. Recent studies showed that obese children have higher urinary KIM-1 levels than healthy controls. Researchers have suggested that KIM-1 is a potential screening biomarker for the detection of early renal injury in obese children [26,34]. Furthermore, urine KIM-1 level has been shown to increase independent of albumin and to serve as a marker of renal tubular injury in the early period in Type 2 diabetic patients [35]. However, the number of studies on obesity and KIM-1 level is low. In the present study, serum and urinary KIM-1 levels were not significantly different between the obese and non-obese groups. Microalbuminuria, which is used to identify early-stage renal injury, is common in obese children and adolescents with a reported prevalence of 10%, and it has been shown to have a strong positive relation with abnormal glucose metabolism and insulin resistance [36,37]. In the present study, microalbuminuria was observed in eight children. In our study, serum and urinary KIM-1 levels were not different in the eight obese children in whom we identified microalbuminuria. A study by Göknar et al. showed that urine KIM-1 levels are increased in hypertensive obese children [26]. In our study, only three of the obese patients had hypertension. The absence of a difference between urinary and serum KIM-1 levels in both groups in our study is attributed both to the small number of patients in our study and to the low rate of obesity-related metabolic and hypertensive complications.

Matrix metalloproteinases (MMPs) are a family of zinc-dependent proteinases with activities that support the degradation and turnover of extracellular matrix (ECM) proteins. An increased circulating concentration of MMP-9 has been demonstrated in patients with obesity, metabolic syndrome, and type 2 diabetes mellitus (T2DM) [38,39]. Plasma MMP-9 level has been reported as a possible biomarker for the development of vascular complications in T2DM [40,41]. In the present study, serum and urinary MMP-9 levels did not differ significantly between the obese and non-obese groups.

Obesity is the main risk factor for the development of insulin resistance, type 2 diabetes and related complications. Chronic low-grade inflammation has been identified as an important component of adipose tissue expansion in obesity. Osteopontin (OPN) is a critical regulator of adipose tissue inflammation. This multifunctional protein is widely distributed in many tissues and body fluids (including plasma, urine, milk, and bile). It mainly regulates bone metabolism and remodeling. Additionally, OPN is involved in pathophysiological processes, such as malignancy, insulin resistance, T2DM, atherosclerosis, end-stage kidney failure, obesity-induced inflammation, and osteoporosis [42,43]. The harmful effect of OPN on the kidneys has been examined in animal studies. Rat and mouse models of diabetic nephropathy showed that OPN was highly expressed in the tubular epithelium of the renal cortex and glomeruli [44]; this was associated with extensive macrophage accumulation in the kidney interstitium, indicating that OPN upregulation and macrophage recruitment may have roles in tubulointerstitial injury in diabetic nephropathy. Consistently, mice are protected from diabetes-induced albuminuria and renal damage, possibly by modulating podocyte signaling and motility [45]. In human studies, plasma OPN level is independently associated with the presence and severity of diabetic nephropathy [11]. In the present study, urinary osteopontin levels significantly increased in the obese participants. Our study results are supported that increased urinary osteopontin level is compatible with AKI in early period of pediatric obesity.

Cystatin C has been accepted a good biomarker to assess GFR in overall population. While Cystatin C has been stated to be uninfluenced by sex, age, or muscle mass, it has now been associated with age, male gender, greater height and weight, higher BMI [46,47]. A strong correlation between cystatin C concentration and end-stage renal disease was demonstrated in morbidly obese patients [48]. Vida Hashemi et al. reported that, while serum creatinine and GFR did not alter significantly, the level of cystatin C increased in obese group when compared lean subjects [49]. In our study, although the levels of serum creatinine and creatinine-based eGFR which is calculated by Schwartz formula [15] were not significantly different, cystatin C level was significantly higher in the obese group when compared to the non-obese group. In this present study these results are compatible with previous study.

Few studies have evaluated renal functions with biomarkers in obese children. The increase in cystatin C in the early stage of obesity without the decreased creatinine-based GFR is significant in terms of the ability of cystatin C to be used as an early marker in the evaluation of renal functions in obese children.

The strength of this study is that it has age-matched control group and comprehensive clinical and laboratory parameters. The limitations of this study are that it is cross-sectional and has a small number of population. It can be carried out in a larger obese children group and as a longitudinal cohort.

## Conclusions

Therefore, we conclude that a panel of serum biomarkers may not be reliable for assessing renal tubular injury in obese children. Importantly, cystatin C seem to be a good marker to assess GFR alteration in the early stages in the obese children. Urinary osteopontin and NGAL levels can be used to determine as noninvasive biomarkers of obesity related AKI in pediatric age groups. Larger prospective studies are necessary to validate the efficacy of these biomarkers for evaluating renal condition in obese group.
